# Detection of *Mycobacterium tuberculosis* multiple strains in sputum samples from patients with pulmonary tuberculosis in south western Uganda using MIRU-VNTR

**DOI:** 10.1038/s41598-022-05591-3

**Published:** 2022-01-31

**Authors:** Lisa Nkatha Micheni, Kennedy Kassaza, Hellen Kinyi, Ibrahim Ntulume, Joel Bazira

**Affiliations:** 1 Department of Microbiology, Mbarara University of Science and Technology, Box 1410, Mbarara, Uganda; 2 Department of Microbiology and Immunology, Kampala International University Western Campus, Box 71, Bushenyi, Uganda; 3 Department of Biochemistry, School of Medicine, Kabale University, Box 317, Kabale, Uganda

**Keywords:** Microbiology, Diseases, Medical research

## Abstract

Infections with multiple strains of *Mycobacterium tuberculosis* are now widely recognized as a common occurrence. Identification of patients infected with multiple strains provides both insight into the disease dynamics and the epidemiology of tuberculosis. Analysis of Mycobacterial Interspersed Repetitive Unit-Variable-Number Tandem Repeats (MIRU-VNTR) has been shown to be highly sensitive in detecting multiple *M. tuberculosis* strains even in sputum. The goal of this study was to identify cases of multiple *M. tuberculosis* strain infections among patients diagnosed with pulmonary tuberculosis in Southwestern Uganda and assessment of factors associated with multiple strain infections. DNA extracted directly from 78 sputum samples, each from an individual patient, was analyzed using the standard 24 loci MIRU-VNTR typing. Five (6.4%) of the 78 patients were infected with multiple strains of *M. tuberculosis* with all of them being the newly diagnosed cases while two-thirds of them were co-infected with HIV. Exact regression analysis projected that the natives were more likely to harbor multiple strains (OR; 0.981, 95% CI 0–7.926) as well as those with a high microbial load (OR; 0.390, 95% CI 0–3.8167). Despite these findings being not statistically significant due to the small sample size, this points to a critical component of disease dynamics that has clinical implications and emphasizes a need for a study using a larger cohort. It is also essential to study the potential factors associated with higher risk of exposure to newly diagnosed and HIV positive patients at the community level. In addition, our ability to detect multiple *M. tuberculosis* strains using the standard 24 loci MIRU-VNTR typing especially with allelic diversity in loci 2059 and 3171, which are excluded from the 15-locus MIRU-VNTR, lead us to recommend the use of this genotyping technique, especially in areas with tuberculosis endemicity similar to this study.

## Introduction

Tuberculosis (TB), caused by members of the *Mycobacterium tuberculosis* complex (MTBC), is one of humanity's oldest scourges and one of the leading causes of death from a single infectious agent globally, accounting for about 1.5 million fatalities and 10 million new cases each year^1–3^. These cases are as a result of either a primary infection, an endogenous reactivation of a primary infection or exogenous reinfection with a new strain^4^. Historically, it was presumed that TB was as a result of a single strain and any recurrence was due to reactivation of the same strain that caused the first episode^5^. Infection due to multiple strains at a single point in time was hardly considered. However, in the mid-1970s using phage typing, it was demonstrated that different strains of MTBC can infect a patient at the same time^6^ either as a result of a single transmission involving multiple distinct strains or due to multiple transmission events^7^. Multiple strain infections can either be due to mixed infections or clonal diversity and drawing clear distinctions between the two in clinical settings is somehow difficult. However, there is a significant difference in how these two mechanisms generate within-host diversity. Clonal diversity involves sporadic polymorphism resulting from sequential adaptive mutations (microevolutions)^8–10^, whereas mixed infection involves a host acquiring an entirely new MTBC genome through successive or concurrent exposure to different strains^11,12^. Considering that members of MTBC have highly conserved genomes^13,14^, high quality methods are required to identify small alterations within the infecting mycobacterial population. So far, various Polymerase Chain Reaction (PCR) based approaches have been utilized to demonstrate multiple strains within the same sputum sample^10,15^ or different sputum samples from the same patient^8,16^. Mycobacterial Interspersed Repetitive Units-variable Number of Tandem Repeats (MIRU-VNTR) analysis, initially developed in 2001^17^, identifies such changes in the genome by varying the copy of repeats in highly variable regions of the MTBC genome^18,19^. This method was found to be adequate for large-scale prospective studies due to its short turnaround time but still lacked the discriminatory power required for long-term, population-based studies in order to account for a large number of samples and recent strain evolution. However, in 2006, Supply et al. proposed an expanded set of 24 MIRU loci that has high discriminatory power and thus recommended for phylogenetic studies^20^. This study aimed at identifying multiple MTBC strain infections among patients with pulmonary tuberculosis (PTB) in a high TB incidence area using MIRU-VNTR analysis and determining factors that could be associated with mixed strains infection in this area. TB incidence in Southwestern Uganda is high at 253 cases per 100 000 people per year^21,22^. It has been shown that multiple MTB strain infections are more common among people living in high TB burdened areas^4,7,11,23^ and accurate identification of this condition provides not only insight into the disease trends but also helps in the management and control of TB^16,24–27^.

## Results

### Prevalence of multiple strains infection

**Figure 1:**
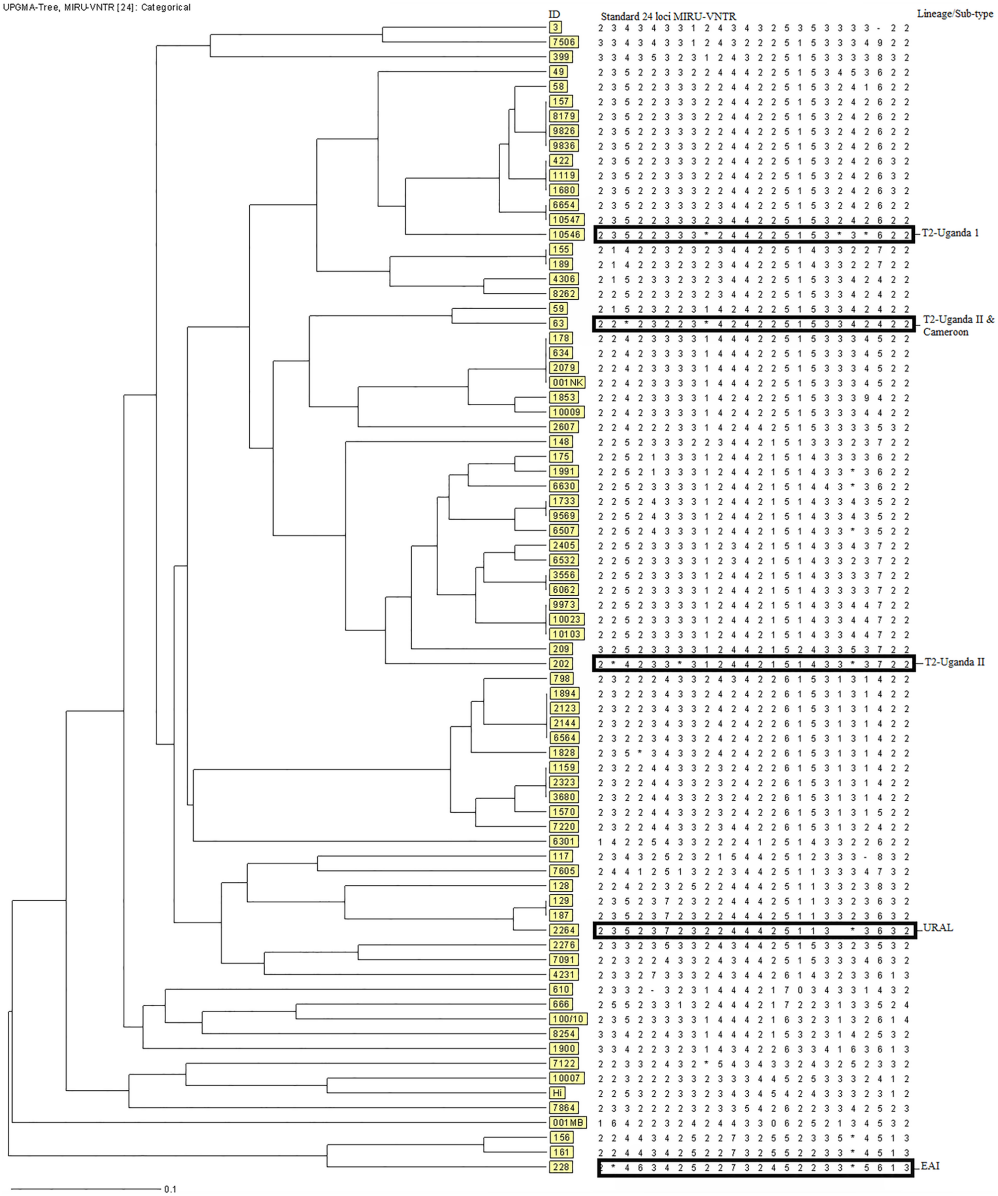
UPGMA tree based on the standard 24 loci MIRU-VNTR of MTB recovered from PTB patients in Southwestern, Uganda. Lineage sub-type identified by MIRU-VNTR*plus* similarity search.

MIRU-VNTR typing was performed on 78 sputum samples, each from an individual PTB patient. Majority of these samples (91%;71/78) were from newly diagnosed cases while 9% (7/78) were relapse patients. Ten (12.8%) patients were from refugees residing in the resettlement camps, 6 (7.7%) were from patients in prison. According to the HIV status records, 39.7%, 24.4%, and 35.9% were HIV positive, negative or unknown respectively. Five of the 78 patients (6.4%; 95% CI 0.864–0.976) were found harbor more than one strain of *M. tuberculosis* with all cases of patients infected with multiple strains being the newly diagnosed patients (p, 0.468) whereas three of them were HIV positive (see Tables 1 and 2). Two of the five samples showed double alleles at three loci (patient # 202 and # 10,546), while two other samples had three alleles in 1 locus and two alleles at another locus (patient # 228 and # 2264) with allelic diversity being noted in loci 424, 1644, 2059, 3171, 3192 and 3690 (see Table 2). Based on the unweighted pair group method with arithmetic mean (UPGMA) analysis of the standard 24 loci MIRU-VNTR, a total of 12 different strains with 58 unique patterns were identified; Uganda I, Uganda II, EAI, LAM, Haarlem, Cameroon, Ghana, URAL, TUR, S, Bovis and Caprae. Six (7.7%) samples did not match any strain in the database hence regarded as unique. Uganda I and Uganda II sub-lineages accounted for 23.1% and 19.2% of the strains respectively whereas Ghana, Tur and S strains were individually identified in 1.3% of the samples. Animal strains; *M. bovis* and *M. caprae* were present in 2.6% and 1.3% of the samples (Table 3). MIRU-VNTR*plus* similarity search indicated that four of the patients with multiple strains had two distinct strains while only one patient (#2264) had three strains. Furthermore, one of the patients (#63) with two distinct strains these strains belonged to two distinct sub-lineages whereas the rest of the patients had strains belonging to the same sub-lineage (Fig. 1). Patient #202 harbored strains that were resistant to isoniazid that had mutations in both katG and inhA regions (Table 2).

**Table 1:** Prevalence of *M. tuberculosis* multiple infections and comparison between patients with multiple versus single strain infections in Southwestern, Uganda.

*Variable*	Category	Patient’s characteristics n (%)	Single Strain (n = 73; 93.6%); 95% CI (0.864–0.976)	Multiple strains (n = 5; 6.4%); 95% CI (0.024–0.136)	χ^2^ p-value
Age	18–24	57 (73.1)	53 (93.0)	4 (7.0)	0.799
	25–44	15 (19.2)	14 (93.3)	1 (6.7)	
	45–64	6 (7.7)	6 (100)	0 (0)	
Gender	Male	63 (80.8)	60 (95.2)	3 (4.8)	0.223
	Female	15 (19.2)	13 (86.7)	2 (13.3)	
HIV status	Positive	31 (39.7)	28 (90.3)	3 (9.7)	0.616
	Negative	19 (24.4)	18 (94.7)	1 (5.3)	
	Unknown	28 (35.9)	27 (96.4)	1 (3.6)	
Level of Income	High	7 (9.0)	7 (100)	0 (0.0)	0.468
	Low	71 (91.0)	66 (93.0)	5 (7.0)	
TB in the past	No	71 (91.0)	66 (93.0)	5 (7.0)	0.468
	Yes	7 (9.0)	7 (100)	0 (0)	
Refugee status	No	68 (87.2)	63 (92.6)	5 (7.4)	0.375
	Yes	10 (12.8)	10 (100)	0 (0.0)	
Imprisoned	No	72 (92.3)	67 (93.1)	5 (6.9)	0.505
	Yes	6 (7.7)	6 (100)	0 (0.0)	
PTB diagnostic results	High/ > 10AFB/OIF	27 (34.6)	24 (88.9)	3 (11.1)	0.123
	Medium/1–10 AFB/OIF	12 (15.4)	10 (83.3)	2 (16.7)	
	Low/10–100AFB/100 OIF	22 (28.2)	22 (100)	0 (0.0)	
	Very low/1–9AFB/100 OIF	17 (21.8)	17 (100)	0 (0.0)	
Rif resistance	No	63 (80.8)	58 (92.1)	5 (7.9)	0.259
	Yes	15 (19.2)	15 (100)	0 (0.0)	
Inh resistance	No	63 (80.8)	67 (94.4)	4 (5.6)	0.373
	Yes	15 (19.2)	6 (85.7)	1 (14.3)	

Statistical significance considered at p-value ≤ 0.05; *OIF* oil immersion field, *Rif* Rifampicin, *Inh* Isoniazid.

**Table 2:** Patients in south western Uganda harboring more than one strain of *M. tuberculosis* identified using MIRU-VNTR standardized 24 loci.

ID	Patient variable		MIRU-VNTR loci																							
N	Case	HIV	1	2	3	4	5	6	7	8	9	10	11	12	13	14	15	16	17	18	19	20	21	22	23	24
63	Nd	+	2	2	4,5	2	3	2	2	3	1,2	4	2	4	2	2	5	1	5	3	3	4	2	4	2	2
202	Nd	+	2	2,3	4	2	3	3	2,3	3	1	2	4	4	2	1	5	1	4	3	3	2,3	3	7	2	2
228	Nd	+	2	2,3,4	4	6	3	4	2	5	2	2	7	3	2	4	5	2	2	3	3	2	2,5	6	1	3
2264	Nd	-	2	3	5	2	3	7	2	3	2	2	4	4	4	2	5	1	1	3	2,3,4	2,3	3	6	3	2
10,546	Nd	Un	2	3	5	2	2	3	3	3	1,2	2	4	4	2	2	5	1	5	3	2,3	3	2,5	6	2	2

The numerical figures represent the number of alleles per amplified MIRU-VNTR loci, as described by Supply et al.^28^. Where there is more than one numerical figure per locus indicates existence of more than one MTB strain in the sample, signifying an infection with multiple strains of *M. tuberculosis*. MIRU-VNTR Loci: 1 = 154, 2 = 424, 3 = 577, 4 = 580, 5 = 802, 6 = 960, 7 = 1644, 8 = 1955, 9 = 2059, 10 = 2163b, 11 = 2165, 12 = 2347, 13 = 2401, 14 = 2461, 15 = 2531, 16 = 2687, 17 = 2996, 18 = 3007, 19 = 3171, 20 = 3192, 21 = 3690, 22 = 4052, 23 = 4156, 24 = 4348. *ID* patient identification number, *Nd* Newly diagnosed, *Un* unknown.

**Table 3:** Distribution of MTB lineages.

Patients, N = 78	
Lineage/strain	Number (%)
Uganda I	18 (23.1)
Uganda II	15 (19.2)
LAM	12 (15.4)
Cameroon	10 (12.8)
URAL	5 (6.4)
EAI	3 (3.8)
Haarlem	3 (3.8)
Bovis	2 (2.6)
Caprae	1 (1.3)
TUR	1 (1.3)
S	1 (1.3)
Ghana	1 (1.3)
Unique	6 (7.7)

### Exact regression analysis of factors associated with multiple strains infection

The conditional maximum likelihood from the bivariable exact regression revealed that none of the patients’ demographic variables such as age, sex was linked to multiple strain infections (Table 4). The model projected that patients who tested with high**/ > **10AFB/OIF (OR 0.390, 95% CI 0–3.8167) and medium/1–10 AFB/OIF (OR; 0.300, 95% CI 0–2.9200) MTB loads in their sample were more likely to be diagnosed with multiple strains compared to those who had tested with Very low/1-9AFB/100 OIF when all other factors were held constant. Furthermore, it was also revealed that the natives were more likely to harbor multiple strains (OR; 0.981, 95% CI 0–7.926) as compared to the refugees. However, because the number of refugees and individuals with multiple strain infections is so small, drawing firm inferences from these findings is difficult.

**Table 4:** Exact bivariate logistic regression of factors associated with multiple strains of *M. tuberculosis* infections among PTB patients in Southwestern, Uganda.

Characteristics	Odds ratio	95% CI	p-value
**Age group (years)**			
18–24	1.780	0–16.388	1.000
25–44	0.947	0.018–10.629	1.000
45–64	1.000		
**Sex**			
Female	3.020	0.231–29.260	0.488
Male	1.000		
**HIV status**			
Positive	2.843	0.213–157.389	0.691
Negative	1.487	0.018–122.040	1.000
Unknown	1.000		
**Level of income**			
Low	1.483*	0–12.483	1.000
High	1.000		
**Incarceration**			
No	1.767*	0–15.236	1.000
Yes	1.000		
**Refugees**			
No	0.981*	0–7.926	1.000
Yes	1.000		
**TB in the past**			
No	1.483	0–12.483	1.000
Yes	1.000		
**PTB diagnostic results**			
High/ > 10AFB/OIF	0.390	0–3.8167	0.4417
Medium/1–10 AFB/OIF	0.300	0–2.9200	0.3175
Low/10-100AFB/100 OIF	1.580	0.1154–16.1333	0.9880
Very low/1-9AFB/100 OIF	1.000		
**Rif**			
No	0.598*	0–4.693	0.666
Yes	1.000		
**Inh**			
No	2.740	0.049–34.662	0.767
Yes	1.000		

*Median unbiased estimates (MUE); *OIF* oil immersion field, *Rif* Rifampicin, *Inh* Isoniazid.

## Discussion

Multiple strain infections in TB are now recognized as common occurrences and identifying patients with multiple MTB strains is critical in clinical practice, public health and molecular epidemiology. This is because not only does it provide insight into the disease patterns but also aids in the management and control of TB. This study revealed that one out of every sixteen PTB patients (6.4%) was infected with multiple strains of MTB. This prevalence is almost similar to the 7.1% reported in Kampala, Uganda^11^ but much lower than the 11% observed in Mubende, Uganda^23^. The disparities in estimations are probably due to discrepancies in the sensitivities of the genotyping techniques used to differentiate between MTB strains. While there are diverse genotyping approaches employed in the identification of mixed infections, the degree of sensitivity of each method varies. While the Mubende and Kampala investigations used 15 loci MIRU-VNTR typing, this study utilized 24 loci MIRU-VNTR typing with a single target conventional PCR. This method has been demonstrated to be very sensitive and discriminative, rendering it the gold standard in the diagnosis of multiple strain infections^29,30^. Other significant discrepancies can be a result of the laboratory methods utilized. As with any other genotyping approaches, detection of multiple strains can only be established when there are sufficient DNA copies of that strain in the sample being studied. Many studies, including the Kampala and Mubende studies, use culture to increase the mycobacterial population^11,23,31^, however, the culture step can drastically change the clonal composition thus influencing the frequency with which multiple strains are detected^9,29,32^. In our study, we utilized DNA isolated directly from processed sputum samples. Detection of multiple strains directly from sputum samples has successfully been documented^29^. However, Our findings are also much higher than the 2.8%^12^ reported in Malawi and 3.2% in Zambia^33^ but lower than the 9.6%^34^ and 10%^35^ reported in Botswana. Differences between the study settings may partly account for this difference whereby for instance the annual risk of TB infection in Malawi is approximately 1%^3,36^ while in Botswana it is 3%^37^.

Our study also revealed that unlike the relapse patients, who were not infected with multiple strains, all (100%) of the patients in our study with multiple strain infections were newly diagnosed cases. This is consistent with the findings of the Mubende study^23^, which observed that the majority (87.5%) of patients with multiple strains were newly diagnosed cases. This might reflect a high level of transmission and heterogeneity of strains in this category of patients (Cohen et al., 2012). This hypothesis is supported by the proportion of newly diagnosed cases that exhibited the multiple strain infection phenomena, an attribute that is reported to indicate high transmission rates^38–40^. Furthermore, a third (9.7%) of the patients with multiple strain infections were also HIV-infected. This finding is consistent with other studies, in which nearly all multiple strain TB infected people were HIV positive^11,23^. This appears to support the notion of the link between multiple strain TB infection and HIV/TB co-infection^16,23,34,41,42^. Given the high prevalence of HIV and HIV/TB co-infection in this region^43^, it is plausible to suggest that HIV-induced immune deficiency exposes patients to the risk of concurrent infections. HIV removes the security of being reinfected as one battles an ongoing infection thereby creating a scenario where one can be infected even before they clear an ongoing infection (Elizabeth Glaser Foundation, 2015).

### Study limitations

A notable limitation of this study is the small sample size of patients with multiple strains which may have limited the precision with which we could estimate the relationship between the feature of interest and the likelihood of outcome/exposure However, exact logistic regression was selected to make such estimates since this type of analysis provides the highest chance of an event occurring within the sub-population formed by the various factors included in the model. Another limitation of this study is that since genotyping was done on genomic DNA extracted directly from MTB positive sputum samples, low levels of DNA could have been obtained especially from samples with very low/trace Mtb load thus insufficient DNA to detect multiple strains. However, doing PCR on isolates is associated with a drastic change of clonal composition^9,29,32^.

## Conclusion

The findings of this study reveal that the rate of multiple strains infection in SWU is at 6.4% with all the patients being diagnosed with TB for their first time having this condition and two-third of them being HIV positive. Despite these findings being not statistically significant due to the small sample size, this points to a critical component of disease dynamics that has clinical implications thus emphasizing a need for a study using a larger cohort. It is also essential to establish the potential factors associated with the high risk of exposure to newly diagnosed patients at the community level. Our ability to detect multiple *M. tuberculosis* strains using the standard 24 loci MIRU-VNTR typing with allelic diversity in loci such as 2059 and 3171, which are excluded from the 15-locus MIRU-VNTR, lead us to recommend the use of this genotyping technique, especially in areas with M. tuberculosis endemicity similar to that observed in this study.

## Materials and methods

### Ethical consideration

This study was approved by the Institutional Review Board of Mbarara University of Science and Technology (MUST-IRB), the Uganda National Council for Science and Technology (with UNCST reference number HS 2379). The health facility administrators and the prime minister granted permission to access their facilities and refugee camps, respectively. Written informed consent was obtained from each patient who participated in the study.

### Patients, sample collection and processing

All methods of this study were carried out in accordance with the approved guidelines. Sputum samples evaluated in this study were from individuals involved in an ongoing epidemiological study in Southwestern, Uganda, from which some papers have been published^44,45^. Sputum samples were collected between May 2018 and April 2019 from patients seeking health care services at either Nakivale HC111, Kabale Regional Referral Hospital and Mbarara Regional Referral Hospital who consented to the study after filling out an informed consent form. The patients were over the age of 18 years who were diagnosed with PTB using either smear microscopy or Cepheid GeneXpert and reported to have not undergone TB treatment in the preceding month.

### DNA extraction

The DNA was extracted directly from clinical sputum samples using the cetyltrimethylammonium bromide (CTAB)-based method described in CLSI^46^ with minor modifications since these were sputum already processed for GeneXpert analysis. The, ZN microscopy-diagnosed samples underwent similar processing as the cepheid Gene Xpert-diagnosed samples by adding the Gene Xpert MTB/RIF sample reagent (Cepheid, Sunnyvale, CA, USA) to the sputum at a 2:1 ratio. The mixture was then manually agitated twice during a 15-min room temperature incubation period. Briefly, sputum samples (containing the GeneXpert processing fluid) were centrifuged for 15 min at 14,000 rpm and the supernatant discarded. The pellet was resuspended in 400μL of TE buffer before being heat-killed at 95 °C for 30 min. Subsequently, 50μL of lysozyme (10 mg/mL in Tris–EDTA [TE] buffer) was added to the sample which was then incubated overnight at 37 °C. After incubation, 70% of sodium dodecyl sulfate and 5μL of proteinase K (20 mg / mL) were added, and the sample incubated at 65 °C for 15 min. One hundred microliters of the pre-heated CTAB / NaCl solution mixture (prepared by mixing 10% CTAB with 0.7 M NaCl and heated at 65 °C for 30 min) was added to the sample in a tube together with 100μL of 5 M NaCl and gently mixed by pipetting. The mixture was then incubated for 10 min at 65 °C, after which 750μL of chloroform: isoamyl alcohol (24:1) was added and gently swirled. The mixture was then centrifuged for 5 min at 14,000 rpm after which the aqueous layer was carefully transferred to a new microcentrifuge tube containing 450μL ice-cold isopropanol and incubated for 30 min at − 20 °C. The mixture was then centrifuged for 15 min at 14,000 rpm and the supernatant discarded. The sample was then rinsed with 70% ice-cold ethanol and centrifuged for 5 min at 14,000 rpm following which ethanol was removed and the sample allowed to air dry for approximately 15 min. The pellet was then resuspended in 50μL TE buffer and incubated at 65 °C to allow resuspension after which the sample was then ready for downstream use.

The quantity of the extracted DNA was obtained using NanoDrop 3300 Fluorospectometer (ThermoFisher Scientific) after which all the samples were confirmed as MTB by PCR-detection of a 123 bp fragment of the IS*6110*, which is common in the members of the MTB complex. Drug susceptibility testing to screen for rifampicin and isoniazid was carried out using high resolution melting curve analysis as described in Micheni et al.^45^.

### Single nucleotide polymorphic (SNP) typing

SNP typing was then performed on these samples using lineage 3 and 4 markers as earlier described in Micheni et al.^44^ to screen for the commonly occurring MTBC lineages in this region while SNP typing was performed as previously described^44^ using lineage 3 and 4 specific primers (Rv004C for MTB L4-U, Rv2962C for MTB L4-NU and Rv0129C for MTB L3) and their accompanying hybridization probes. Briefly, the assays were performed in 20 µl reaction mixture containing 3.75 μl of PCR water, 1.25 μl (0.5 μM final concentration) of each primer, 0.625 μl (0.25 μM final concentration) of each probe, 9.5 μl of 2X Lunar^®^ Universal genotyping master mix, and 3 μl (5–50 ng) of extracted genomic DNA. RT-PCR was carried out in a B*io-Rad CFX96* Touch™ that was programmed for PCR amplification and a melting curve stage. For each of the three uniplex assays, the amplification stage consisted of a pre-PCR stage performed at 95 °C for 10 min, an amplification stage with denaturation at 95 °C for 30 s, primer annealing (50 °C for Rv004C or 52 °C for Rv0129C or 51 °C for Rv2962C) for 30 s and extension at 60 °C for 30 s for 45 cycles. The melting curve analysis consisted of denaturation of the amplicons at 95 °C for 10 s to produce single-stranded DNA, probe annealing temperature at 65 °C for 05 s with a continuous acquisition mode to allow capture the fluorescence and probe melting temperature ranging from 40–80 °C. The MTBC lineages were identified based on differences in melting temperature (Tm)*.* H37Rv (L4-NU), *kc*32969 (L4-U) and delicus (L3) genomic DNA were used as positive control while non-template mix as a negative control.

### MIRU-VNTR typing

The MIRU-VNTR PCRs were performed on genomic DNA extracted from the sputum samples using primers specific for sequences flanking the MIRU units (see Table 5). The PCR was designed to amplify a standard set of 24 MIRU-VNTR loci from genomic DNA retrieved from each sample. Each MIRU locus was amplified individually using a reaction mix and amplification profile described by Supply^28^ with slight modifications. Briefly, assays for the various simplex reactions were prepared according to Supply (2005). Two microliters (5–50 ng) of extracted genomic DNA was added to the PCR pre-mix and amplified in the MultiGene™ OptiMax Thermal Cycler (Massachusetts, USA) which was programmed for PCR amplification with a pre-PCR stage performed at 95 °C for 15 min, an amplification stage with denaturation at 94 °C for 60 s, primer annealing at 59 °C for 60 s, extension at 72 °C for 90 s for 40cycles and a final extension at 72 °C for 10 min. In all the assays, *M. tuberculosis* H37Rv was used as positive controls and sterile water as the negative control. Ten microliters of each PCR product were separated electrophoretically on 2% agarose gels for 3 h, with a 100-bp DNA ladder (Solis Biodyne™, Estonia) serving as size markers. The corresponding MIRU-VNTR bands in the gel images were reported as Roman numerals representing the number of repeats per loci as described in the protocol reference table by Supply (2005)^28^. For any sample that revealed multiple bands at any of the MIRU loci, the PCR was repeated to confirm the results. Multiple strains were concluded as being present if a sample had double alleles at more than one locus while those samples that had varying copy numbers at a single locus were considered as single strain evolution rather than multiple strains.

**Table 5:** PCR primer sequences and MIRU-VNTR locus designations^1^ used in this study.

Loci	Alias	Repeating unit length (bp)	Primer sequences (5’-3’)
580	MIRU4, ETRD	77	GCGCGAGAGCCCGAACTGC GCGCAGCAGAAACGTCCAGC
2996	MIRU26	51	CCCGCCTTCGAAACGTCGCT TGGACATAGGCGACCAGGCGAATA
802	MIRU40	54	GGGTTGCTGGATGACAACGTGT GGGTGATCTCGGCGAAATCAGATA
960	MIRU10	53	GTTCTTGACCAACTGCAGTCGTCC GCCACCTTGGTGATCAGCTACCT
1644	MIRU16	53	TCGGTGATCGGGTCCAGTCCAAGTA CCCGTCGTGCAGCCCTGGTAC
3192	MIRU31, ETR E	53	CTGATTGGCTTCATACGGCTTTA GTGCCGACGTGGTCTTGAT
424	Mtub04	51	GTCCAGGTTGCAAGAGATGG GGCATCCTCAAACAACGGTAG
577	ETR C	58	GACTTCAATGCGTTGTTGGA GTCTTGACCTCCACGAGTGC
2165	ETR A	75	ATTTCGATCGGGATGTTGAT TCGGTCCCATCACCTTCTTA
2401	Mtub30	58	AGTCACCTTTCCTACCACTCGTAAC ATTAGTAGGGCACTAGCACCTCAAG
3690	Mtub39	58	AATCACGGTAACTTGGGTTGTTT GATGCATGTTCGACCCGTAG
4156	QUB-4156	59	TGACCACGGATTGCTCTAGT GCCGGCGTCCATGTT
2163b	QUB-11b	69	CGTAAGGGGGATGCGGGAAATAGG CGAAGTGAATGGTGGTGGCAT
1955	Mtub21	57	AGATCCCAGTTGTCGTCGTC CAACATCGCCTGGTTCTGTA
4052	QUB-26	111	GGCCAGGTCCCTCCCGAT AACGCTCAGCTGTCGGAT
154	MIRU 2	53	TGGACTTGCAGCAATGGACCAACT TACTCGGACGCCGGCTCAAAAT
2531	MIRU 23	53	CAGCGAAACGAACTGTGCTATCAC CGTGTCCGAGCAGAAAAGGGTAT
4348	MIRU 39	53	CGCATCGACAAACTGGAGCCAAAC CGGAAACGTCTACGCCCCACACAT
2059	MIRU 20	77	TCGGAGAGATGCCCTTCGAGTTAG GGAGACCGCGACCAGGTACTTGTA
2687	MIRU 24	54	CGACCAAGATGTGCAGGAATACAT GGGCGAGTTGAGCTCACAGAA
3007	MIRU 27, QUB-5	53	TCGAAAGCCTCTGCGTGCCAGTAA GCGATGTGAGCGTGCCACTCAA
2461	ETR B, VNTR 48	57	GCGAACACCAGGACAGCATCATG GGCATGCCGGTGATCGAGTGG
2347	Mtub 29; VNTR 46	57	ATGATGGCACACCGAAGAAC AACCCATGTCAGCCAGGTTA
3171	Mtub 34; VNTR 49	54	GCAGATAACCCGCAGGAATA GGAGAGGATACGTGGATTTGAG

^1^Extracted from Yasmin et al.^27^. Primers were synthesized by Inqaba Biotec (South Africa).

### Statistical analysis

Patients’ biodata and the presence or absence of multiple strain infection results were entered and validated in Microsoft Excel® 2013. The data was then exported to Stata (Stata/SE 14.2 for windows, Stata Corp, College Station, TX) for statistical analysis. Chi-square test was used to compute proportions and determine the relationship between independent factors and dependent variables (presence multiple strain infection) with statistical significance considered at a 95% level of confidence. Since the feature of interest (multiple strain infections) was found in a small number of patients, an exact bivariate logistic regression analysis was performed to obtain odds ratios for factors that could be associated with the occurrence of multiple strains of *M. tuberculosis* among PTB patients in our setting. Exact logistic regression was selected because it calculates the conditional maximum chance of an event occurring within the sample population described by the model's varying factors. We did not specify a statistical significance threshold as per the recent statistical guidelines^47,48^.

## References

[1] WHO (2016). Global Tuberculosis Report 2016.

[2] WHO (2018). Global Tuberculosis Report 2018.

[3] WHO (2019). Global Tuberculosis Report 2019.

[4] McIvor A, Koornhof H, Kana BD (2017). Relapse, re-infection and mixed infections in tuberculosis disease. Pathog. Dis..

[5] Stead WW (1967). Pathogenesis of a first episode of chronic pulmonary tuberculosis in man: recrudescence of residuals of the primary infection or exogenous reinfection?. Am. Rev. Respir. Dis..

[6] Bates JH, Stead WW, Rado TA (1976). Phage type of tubercle bacilli isolated from patients with two or more sites of organ involvement. Am. Rev. Respir. Dis..

[7] Tarashi S, Fateh A, Mirsaeidi M, Siadat SD, Vaziri F (2017). Mixed infections in tuberculosis: The missing part in a puzzle. Tuberculosis.

[8] Cohen T, Wilson D, Wallengren K, Samuel EY, Murray M (2011). Mixed-strain Mycobacterium tuberculosis infections among patients dying in a Hospital in KwaZulu-Natal, South Africa. J. Clin. Microbiol..

[9] Martin A (2010). The clonal composition of Mycobacterium tuberculosis in clinical specimens could be modified by culture. Tuberculosis.

[10] Shamputa IC (2006). Mixed infection and clonal representativeness of a single sputum sample in tuberculosis patients from a penitentiary hospital in Georgia. Respir. Res..

[11] Dickman KR (2010). Detection of multiple strains of Mycobacterium tuberculosis using MIRU-VNTR in patients with pulmonary tuberculosis in Kampala. Uganda. BMC Infect. Dis..

[12] Mallard K (2010). Molecular detection of mixed infections of Mycobacterium tuberculosis strains in sputum samples from patients in Karonga District, Malawi. J. Clin. Microbiol..

[13] Manson AL (2017). Genomic analysis of globally diverse Mycobacterium tuberculosis strains provides insights into the emergence and spread of multidrug resistance. Nat. Genet..

[14] Gagneux S (2013). Genetic diversity in mycobacterium tuberculosis. Curr. Top. Microbiol. Immunol..

[15] Warren RM (2004). Patients with active tuberculosis often have different strains in the same sputum specimen. Am. J. Respir. Crit. Care Med..

[16] Cohen T (2012). Mixed-strain *Mycobacterium tuberculosis* infections and the implications for tuberculosis treatment and control. Clin. Microbiol. Rev..

[17] Supply P (2001). Automated high-throughput genotyping for study of global epidemiology of *Mycobacterium tuberculosis* based on mycobacterial interspersed repetitive units. J. Clin. Microbiol..

[18] Allix-Béguec C, Harmsen D, Weniger T, Supply P, Niemann S (2008). Evaluation and strategy for use of MIRU-VNTRplus, a multifunctional database for online analysis of genotyping data and phylogenetic identification of Mycobacterium tuberculosis complex isolates. J. Clin. Microbiol..

[19] Kremer K (2005). Discriminatory power and reproducibility of novel DNA typing methods for Mycobacterium tuberculosis complex strains. J. Clin. Microbiol..

[20] Supply P (2006). Proposal for standardization of optimized mycobacterial interspersed repetitive unit-variable-number tandem repeat typing of Mycobacterium tuberculosis. J. Clin. Microbiol..

[21] Ministry of Health Uganda (2016). Annual Health Sector performance report 2015/16. Udhs.

[22] Ministry of Health Uganda. *National Tuberculosis and Leprosy Division July 2017 – June 2018 Report*. vol. 48 (2018).

[23] Muwonge A (2013). Molecular investigation of multiple strain infections in patients with tuberculosis in Mubende district, Uganda. Infect. Genet. Evol..

[24] Zetola NM (2014). Clinical outcomes among persons with pulmonary tuberculosis caused by Mycobacterium tuberculosis isolates with phenotypic heterogeneity in results of drug-susceptibility tests. J. Infect. Dis..

[25] Sivanand T (2018). Treatment correlates of successful outcomes in pulmonary multidrug-resistant tuberculosis: an individual patient data meta- analysis. Lancet.

[26] Van Rie A (2005). Reinfection and mixed infection cause changing Mycobacterium tuberculosis drug-resistance patterns. Am. J. Respir. Crit. Care Med..

[27] Kempker RR (2015). Acquired drug resistance in mycobacterium tuberculosis and poor outcomes among patients with multidrug-resistant tuberculosis. Emerg. Infect. Dis..

[28] Supply P (2005). Multilocus variable number tandem repeat genotyping of mycobacterium tuberculosis. Inst. Pasteur Lille.

[29] Farmanfarmaei G (2017). Bias in detection of Mycobacterium tuberculosis polyclonal infection: Use clinical samples or cultures?. Mol. Cell. Probes.

[30] Bouklata N (2015). Molecular typing of mycobacterium tuberculosis complex by 24-locus based MIRU-VNTR typing in conjunction with spoligotyping to assess genetic diversity of strains circulating in Morocco. PLoS ONE.

[31] Stavrum R (2009). High diversity of Mycobacterium tuberculosis genotypes in South Africa and preponderance of mixed infections among ST53 isolates. J. Clin. Microbiol..

[32] Hanekom M (2013). Population structure of mixed mycobacterium tuberculosis infection is strain genotype and culture medium dependent. PLoS ONE.

[33] Mulenga C (2010). Diversity of Mycobacterium tuberculosis genotypes circulating in Ndola. Zambia. BMC Infect. Dis..

[34] Shin SS (2018). Mixed mycobacterium tuberculosis-strain infections are associated with poor treatment outcomes among patients with newly diagnosed tuberculosis, independent of pretreatment heteroresistance. J. Infect. Dis..

[35] Zetola NM (2014). Mixed Mycobacterium tuberculosis complex infections and false-negative results for rifampin resistance by genexpert MTB/RIF are associated with poor clinical outcomes. J. Clin. Microbiol..

[36] Vorkas C (2012). Tuberculosis drug resistance and outcomes among tuberculosis inpatients in Lilongwe, Malawi. Malawi Med. J..

[37] Tuelo M (2019). Genetic diversity of *Mycobacterium tuberculosis* strains circulating in Botswana. PLoS ONE.

[38] Perez-Lago (2015). Co-infection with drug-susceptible and reactivated latent MDR Mtbletter. Emerg. Infect. Dis..

[39] Zamani S (2015). Determination of circulating Mycobacterium tuberculosis strains and transmission patterns among TB patients in Iran, using 15 loci MIRU-VNTR. Int. J. Mycobacteriol..

[40] Tessema B (2013). Molecular epidemiology and transmission dynamics of Mycobacterium tuberculosis in Northwest Ethiopia: New phylogenetic lineages found in Northwest Ethiopia. BMC Infect. Dis..

[41] Cohen T (2016). Within-host heterogeneity of mycobacterium tuberculosis infection is associated with poor early treatment response: A prospective cohort study. J. Infect. Dis..

[42] Shin SS (2015). Advanced immune suppression is associated with increased prevalence of mixed-strain mycobacterium tuberculosis infections among persons at high risk for drug-resistant tuberculosis in Botswana. J. Infect. Dis..

[43] Elizabeth Glaser Foundation. *Strengthening the Tuberculosis and HIV / AIDS Response in the Southwest Region of Uganda ( STAR-SW ) Project*. (2015).

[44] Micheni LN, Kassaza K, Kinyi H, Ntulume I, Bazira J (2021). Diversity of mycobacterium tuberculosis complex lineages associated with pulmonary tuberculosis in Southwestern, Uganda. Tuberc. Res. Treat..

[45] Nkatha L (2021). Rifampicin and isoniazid drug resistance among patients diagnosed with pulmonary tuberculosis in southwestern Uganda. PLoS ONE.

[46] CLSI (2008). Laboratory Detection and Identification of Mycobacteria; Approved Standard.

[47] Greenland S (2016). Statistical tests, P values, confidence intervals, and power: a guide to misinterpretations. Eur. J. Epidemiol..

[48] Wasserstein RL, Lazar NA (2016). The ASA’s statement on p-values: Context, process, and purpose. Am. Stat..

